# Proximate mechanism of behavioral manipulation of an orb-weaver spider host by a parasitoid wasp

**DOI:** 10.1371/journal.pone.0171336

**Published:** 2017-02-03

**Authors:** Thiago Gechel Kloss, Marcelo Oliveira Gonzaga, Leandro Licursi de Oliveira, Carlos Frankl Sperber

**Affiliations:** 1 Programa de Pós-Graduação em Entomologia, Universidade Federal de Viçosa, Viçosa, Minas Gerais, Brazil; 2 Universidade Federal do Espírito Santo, Centro de Ciências Exatas, Naturais e da Saúde, Departamento de Biologia, Alegre, Espírito Santo, Brazil; 3 Instituto de Biologia, Universidade Federal de Uberlândia, Uberlândia, Minas Gerais, Brazil; 4 Departamento de Biologia Geral, Universidade Federal de Viçosa, Viçosa, Minas Gerais, Brazil; University of Vienna, AUSTRIA

## Abstract

Some ichneumonid wasps induce modifications in the web building behavior of their spider hosts to produce resistant “cocoon” webs. These structures hold and protect the wasp’s cocoon during pupa development. The mechanism responsible for host manipulation probably involves the inoculation of psychotropic chemicals by the parasitoid larva during a specific developmental period. Recent studies indicate that some spiders build cocoon webs similar to those normally built immediately before ecdysis, suggesting that this substance might be a molting hormone or a precursor chemical of this hormone. Here, we report that *Cyclosa* spider species exhibiting modified behavior presented higher 20-OH-ecdysone levels than parasitized spiders acting normally or unparasitized individuals. We suggest that the lack of control that spiders have when constructing modified webs can be triggered by anachronic activation of ecdysis.

## Introduction

Parasites and parasitoids often alter specific behaviors of their hosts. Examples of changes range from subtle shifts in one aspect of the host’s behavior to the performance of completely novel and complex behavioral patterns [1]. Some of these changes might be primarily due to the host’s own defensive responses to infection [1,2] or to exploitation of the host’s nutritional resources by the parasites [3]. Others changes in behavior are classified as host manipulation to acquire some benefit in survivorship or dispersal ability [1]. In the majority of manipulation cases, however, little is known about the proximal mechanisms responsible for behavioral alterations [4]. For example, the cricket *Nemobius sylvestris* (Bosc, 1792) performs the unusual behavior of searching for water and jumping into it after being attacked by mature individuals of the hairworm *Paragordius tricuspidatus* (Dufour, 1828). This behavior is induced by the parasite producing protein from the Wnt family, which act on the central nervous system of the host [5]. Another reported case of host manipulation involves the injection of venom containing a dopamine-like substance into the cerebral ganglia of the cockroach *Periplaneta americana* (Linnaeus, 1758) by the parasitoid wasp *Ampulex compressa* (Fabricius, 1781). This action directly stimulates the grooming-releasing circuits within the host’s cerebral ganglia, which enters into a subsequent nonparalytical hypokinetic state [6,7].

Behavioral modifications of spiders by parasitoid wasps that form part of the tribe Ephialtini [8] (Hymenoptera: Ichneumonidae, Pimplinae) have been observed in several host families. The wasps lay one egg on the spider's abdomen, where the ectoparasitic larva develops by sucking the host’s hemolymph through perforated holes in the spider’s cuticle. Usually, the host spider builds a modified web just before the wasp larva enters the final stage of development [9–18], but, in some cases, the number of radii and spirals of the orb web gradually decrease over several days before the spider dies [19,20]. After the spider builds this modified structure, the larva kills its host, and builds its cocoon attached to the silk threads spun by the host. Cocoon webs appear to be more resistant to rupture [18,20,21] and have fewer components (e.g., radii and sticky spirals in orb webs) that are utilized for prey interception and retention. Variations of this general pattern include the addition of barrier threads [22], shorter radii, doubled number of lines in each radius, construction of a reinforced frame [19], reinforcement of the retreat by addition of more threads [14] or a veil sheltering the cavity containing spider and larva [23]. These alterations are not a by-product of infection [20,21], but appear to be directly induced by the parasitoid larva. This hypothesis is supported by the fact that removing the larva after the construction of the modified web results in the spider gradually building webs that progressively resembles normal webs [19].

The exact proximate mechanism of spider manipulation is unknown, however recent evidence has showed that it might be related to the process of ecdysis. Modified webs of *Cyclosa argenteoalba* Bösenberg & Strand, 1906 [18], *Allocyclosa bifurca* (McCook, 1887) [19], and *Nephila clavipes* (Linnaeus, 1767) [20] resemble the molting webs spun by unparasitized individuals just before molting. During the molting period, spiders have increasing levels of the hormone 20-OH-ecdysone (20E), which is responsible for renewing their old exoskeletons [24]. This fact resulted in us hypothesizing that the presence of parasitoid wasp larvae would induce the increase of 20E levels in their spider hosts just before pupation. These higher 20E levels would elicit a behavioral response of the parasitized spiders, to build molting webs. In the present study, we evaluated this hypothesis, by testing whether the that 20E levels in parasitized spiders that already present modified web-building behavior are higher than those in unparasitized spiders, and whether they are higher than parasitized spiders performing unmodified web-building behavior. We also tested a second hypothesis, that 20E levels would be higher in the third stage of the parasitoid larvae, which is one stage that induces the behavioral modification in the spider host, than in the second stage larvae. In addition, we described the structure of molting webs and compared these to normal webs and cocoon webs. We utilized two orb-weaver spiders *C*. *morretes* Levi, 1999 and *C*. *fililineata* Hingston, 1932 (Araneidae) parasitized by the wasps *Polysphincta janzeni* Gauld, 1991 and *P*. sp. nr. *purcelli* (Ichneumonidae), respectively, as models.

## Materials and methods

### Study species

*Cyclosa fililineata* is a small spider (total length of 4.7 mm) that is found from Panama to Argentina. *C*. *morretes* is slightly larger (total length of 7.2 mm) and has only been reported from Brazil [25,26]. The parasitoid of *C*. *morretes*, *P*. *janzeni*, has been previously reported from Costa Rica [27] and the Brazilian Atlantic rainforest [21,28]. The parasitoid of *C*. *fililineata*, *P*. sp. nr. *purcelli*, is probably a new species, with the closely related *Polysphincta purcelli* Gauld, 1991 being reported from Belize and Costa Rica [27,29]. Web modifications of *C*. *morretes* parasitized by *P*. *janzeni* and *C*. *fililineata* parasitized by *P*. nr. sp. *purcelli* have been recently described (see [21]).

### Study area

All spiders/larvae were collected during July 2014 and January 2015 from two Atlantic rainforest reserves located in the Santa Teresa municipality, in Espírito Santo state, Brazil; specifically, Estação Biológica de Santa Lúcia (19°57′56″S, 40°32′24″W) and Reserva Biológica Augusto Ruschi (19°54′26″S, 40°33′11″W).

### Spider and wasp collection

We collected 10 and 6 unparasitized females of *C*. *morretes* and *C*. *fililineata*, respectively. We also collected 11 females of *C*. *morretes* parasitized by second stage larvae of *P*. *janzeni* and 6 females of *C*. *fililineata* parasitized by second stage larvae of *P*. sp. nr. *purcelli* that were in normal webs. Finally, we collected 10 and 4 females of *C*. *morretes* and *C*. *fililineata*, parasitized by third stage larvae of *P*. *janzeni* and *P*. sp. nr. *purcelli*, respectively.

Second stage larvae were classified based on their segmented bodies, while third stage larvae identification was based on the observation of characteristically developed dorsal tubercles, which are absent in preceding developmental stages. However, it is possible that these stages did not correspond exactly to larval instars.

We collected only females because males are rarely parasitized. After collection in the field, all spiders were immediately killed by contact with ice in microtube vials. We separated the larvae from their hosts in the Santa Lucia Station laboratory, and they were immediately frozen at 0°C in microtube vials. The spiders were then transported frozen at 0°C to the Núcleo de Análise de Biomoléculas of the Universidade Federal de Viçosa and kept at -20°C until ecdysone analysis.

All females of *C*. *morretes* were subadults, whereas all females of *C*. *fililineata* were adults that did not have egg sacs in their webs. Individuals of both spider species carrying third stage larvae were collected the day after they had constructed a cocoon web (modified behavior). All individuals of *C*. *morretes* and *C*. *fililineata* built these cocoon webs only in the last day of their lives, when parasitoid larvae reached the third stage characterized by the presence of dorsal tubercles [30].

The interval for larvae to induce behavioral modifications in their spider hosts is between 21 and 24 days [30]. This perfect agreement between the occurrence of host modified behaviors and larval age, apart from the observation of modified behaviors in adult spiders (that do not make molts), indicates that changes in web construction cannot be attributed to hormonal shifts during regular molts.

Additional voucher specimens of *P*. sp. nr. *purcelli* and *P*. *janzeni* specimens were identified by the taxonomist Ana Paula S. Loffredo and deposited in the collection at Universidade Federal de São Carlos (curator, A. M. Penteado-Dias), and specimens of *C*. *morretes* and *C*. *fililineata* were deposited in the arachnid collection of Centro de Coleções Taxonômicas da Universidade Federal de Minas Gerais (curator, A. J. Santos) in Minas Gerais, Brazil.

### Ethic statement

Fieldwork was performed with permission from the System of Authorization and Information on Biodiversity—SISBIO/ICMBio (Authorization No. 34711/1, Brazil) and complied with the current legal and ethical requirements for animal welfare. The species used in the present study were not endangered or protected in Brazil.

### Web structure description

To describe the structure of molting, cocoon, and normal webs, we followed [21]. We quantified structural web changes in the field by first covering the webs with water, then photographing them. We evaluated changes in radii and spiral number, which are variables previously utilized to describe web alterations resulting from spider host and larval parasitoid interactions [20, 21]. Molting webs were identified by the presence of exoskeletons adhered to the webs.

### Ecdysteroid analyses

To analyze the levels of 20E in spiders and larvae, we prepared a stock solution of commercial 20E in methanol, at a final concentration of 5.0 ng/mL. The steroid 20E was obtained from Sigma-Aldrich (St. Louis, MO, USA), with a purity of ≥93%. Methanol was obtained from Vetec Química Fina LTDA (Duque de Caxias, RJ, Brazil). The work standard solutions of 20E, with final concentrations of 0.05, 0.1, 0.5, 0.8, 1.0, and 2 ng/mL, were prepared in methanol and stored at -20°C. Standards were used to construct the calibration curves by plotting the peak height *versus* 20E concentrations.

Spiders and larvae were weighed inside 2 mL microtubes. After weighing, 500 μL of methanol at ambient temperature (25°C ± 2) was added to each microtube, and the samples were homogenized for 3 min using Tissue Master 125 (Omni International) to extract 20E. The sample was centrifuged at 11000 g for 20 min, and the upper methanol layer was transferred to another microtube. Then, the samples were centrifuged again at 11000 g for 20 min, and the upper methanol layer was transferred to injection glass vials and analyzed by liquid chromatography mass spectrometry.

The liquid chromatography mass spectrometry ecdysone analyses were performed with the liquid chromatography instrument UHPLC Agilent Technologies 1290 Infinity (Santa Clara, California, USA) in combination with an Agilent triple quadruple 6430 mass spectrometer with electrospray ionization (Santa Clara, California, USA) (adapted from [31,32]). The system operation was controlled and processed with the software MassHunter B06.00. Chromatographic separation was carried out using the Eclipse Plus C18um column (50 × 2.1 mm) (Agilent Technologies, Santa Clara, California, USA). 20E was separated using binary gradient elution, which mobile phase A was water containing 0.01% acetic acid, and phase B was acetonitrile containing 0.02% acetic acid. Acetonitrile was obtained from Fluka Analytical (Seelze, Germany), acetic acid from Sigma-Aldrich (St. Louis, MO, USA), and pure water was obtained by Milli-Q Direct Water Purification System (Darmstadt, Germany).

The gradient started with 15% phase B up to 1.5 min and then increased to 90% phase B, which was maintained for 6 min. Subsequently, phase B was decreased to 10% and maintained for 7 min. The total run time was 7 min, and an equilibration step of 1 min was included. The flow rate of the mobile phase and the column temperature were set at 0.3 mL/min (injection volume: 10μL) and 30°C, respectively. To avoid carryover effect, the auto sampler needle was rinsed automatically with the high performance liquid chromatography flushing solvent (Agilent Technologies, Santa Clara, California, USA) for 3 s before each analysis.

The mass spectrometry detection of the steroid was conducted by multiple reactions monitoring in the positive ion mode. The tuning parameters optimized for 20E were gas at 300°C, drying gas flow at 3 L/min, nebulizer pressure at 15 psi, and Vcap voltage at 4000 V. The scans for mass spectrometry spectra were acquired in the m/z of 481, which is the precursor ion of 20E. The product ions obtained from this precursor were the ions 371 and 445 m/z in the retention time 1.4 (± 0.05) min. The quantification of the 20E concentration (pg/mL/mg) was obtained by comparing the peak height in each sample with calibration curves, divided by the weight of each sample. We only considered peaks with signal to-noise ratios greater than five, as recommended by food and drug administration guidelines.

### Statistical analyses

For each spider species, we performed one-way analyses of variance to compare 20E concentration of 371 and 445 product ions in spiders parasitized by third stage larvae (already presenting modified behavior), spiders presenting normal behavior, parasitized by second-stage larvae, unparasitized spiders, and their respective second and third-stage wasp larvae. We considered 20E concentration (pg/mL/mg) as the response variable, and spider (parasitized by larvae in the second or third stage and unparasitized spiders) or larva (second or third stage) category, as the explanatory factor, comprehending five factor levels. We fitted generalized linear models with Gamma distribution and canonical reciprocal link function.

We used *a priori* contrasts, in single degree of freedom comparisons, to evaluate the differences in 20E 371 and 445 ion concentrations among the five levels of our explanatory factor: (i) spiders parasitized by larvae of second or (ii) third stage larva; (iii) unparasitized spiders, and parasitoid larvae of (iv) second and (v) third stages. The sequence of the *a priori* amalgamations obeyed the reasoning that we would go from the categories where we expected lowest 20E concentration to the categories were we expected the highest 20E concentrations, based on the hypothesis that 20E was involved in the modification of the web building behavior. First, we tested whether the full model, with five-level explanatory factor, was significantly different from a null model. Once the full model was significant, we proceeded with model simplification, amalgamating the explanatory factor levels according to the following sequence: (i) unparasitized spiders + second stage parasitoid larvae; (ii) unparasitized spiders + second stage parasitoid larvae + spiders parasitized by second stage larva, and (iii) unparasitized spiders + second stage parasitoid larvae + spiders parasitized by second stage larva + third stage parasitoid larvae. A further amalgamation (unparasitized spiders + second stage parasitoid larvae + spiders parasitized by second stage larva + third stage parasitoid larvae + spiders parasitized by second stage larvae) would result in the null model, where the explanatory factor had a single level, i.e., the intercept of the statistical model. Each simplified model was compared to the preceding model. We proceeded with model simplification only up to the first significant difference, using *F* test, that is recommended for Gamma error distributions [33]. Full models and minimum adequate models were checked by residual analysis.

## Results

**Fig 1:**
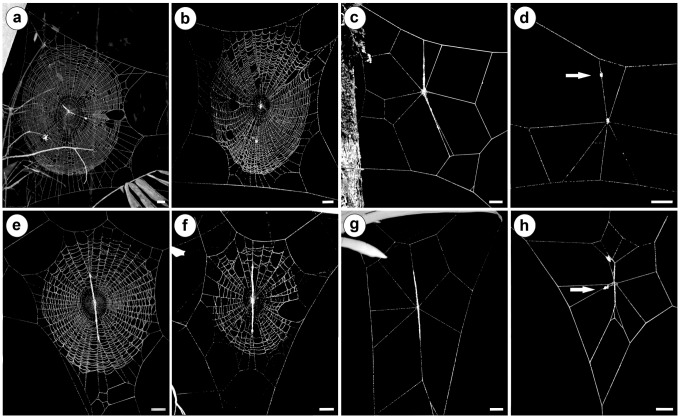
**Webs of *Cyclosa morretes* (a–d) and *Cyclosa fililineata* (e–h) spiders.** Webs of an unparasitized female (a, e); webs of female parasitized by second stage larva (b, f); webs built by female parasitized by third stage larva (c, g); and molting webs of unparasitized spiders (d and h). Arrows point to an old exoskeleton of the web-weaver spider. Scale bars = 1 cm.

For both spiders species, *C*. *morretes* and *C*. *fililineata*, the molting web showed a simplification in its structure, characterized by absence of spirals and reduction in the number of radii to less than ten (*C*. *morretes*: 8.8 ± 0.5, n = 10 and *C*. *fililineata*: 8.0 ± 0.5, n = 5; Fig 1D and 1H). The molting web maintained its modified structure for up to six days (range: 2–6 days; n = 9), while the web-constructing spider remained motionless at the center of the web. During the second day after building the molting web, spiders replaced their exoskeletons.

The molting web structure was similar to the cocoon web, which was observed in spiders parasitized by third stage larvae of *Polysphincta* spp. (*C*. *morrete*s: 7.7 ± 0.2 radii and 0 spirals, n = 15; *C*. *fililineata*: 7.6 ± 0.3 radii and 0 spirals, n = 9; Fig 1C and 1G), and differed from the web structure of nonparasitized spiders, as well as from the web structure of spiders parasitized by second stage larvae. Both spider species presented normal webs with varying numbers of radii and spirals (*C*. *morretes*: 44.8 ± 3.9 radii and 23 ± 2.6 spirals, n = 15; *C*. *fililineata*: 52.5 ± 2.5 radii and 33 ± 2.1 spirals, n = 15) (Fig 1A and 1E). These same web patterns are observed in webs of spiders parasitized by second-stage larvae (*C*. *morretes*: 40.7 ± 3.7 radii and 24.8 ± 1.9 spirals, n = 15; *C*. *fililineata*: 48 ± 2.1 radii and 27.9 ± 2.5 spirals, n = 14; Fig 1B and 1F).

**Fig 2:**
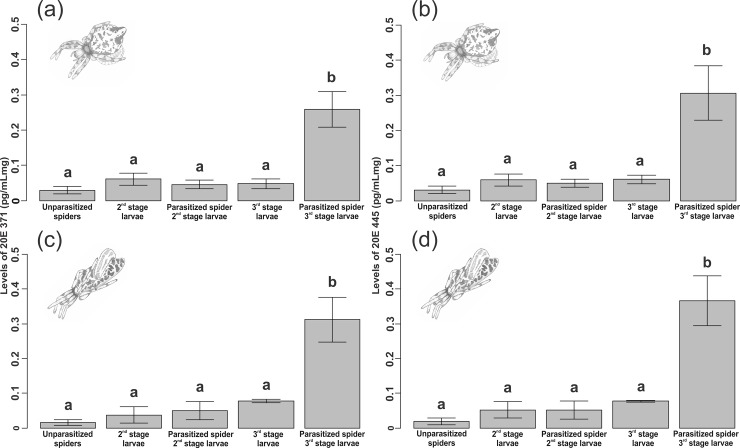
Comparison of levels of 20-OH-ecdysone (20E) in host spiders and parasitoids. Levels of product ion 371 (a) and 445 (b) for *C*. *morretes*/*P*. *janzeni* and levels of product ion 371 (c) and 445 (d) for *C*. *fililineata*/*P*. sp. nr. *purcelli*. Categories follow the *a priori* order of the contrasts. Bars are means, and whiskers are standard errors of the mean. Different lower case letters correspond to significant differences (p < 0.001) among web structure.

For *C*. *morretes*, concentrations of 20E product ion 371 and ion 445 (pg/mL/mg) were higher in spiders parasitized by third stage larva (ion 371: F_1,50_ = 64.81, p < 0.001, Fig 2A, S1 Fig; ion 445: F_1,50_ = 52.85, p < 0.001, Fig 2B, S2 Fig) than in all other analyzed categories. All remaining categories were similar, as follows: there were no differences in the concentrations of both 20E product ion 371 and ion 445 among unparasitized spiders and second stage larva (ion 371: F_1,47_ = 1.01, p = 0.32; ion 445: F_1,47_ = 0.49, p = 0.48), spiders parasitized by second stage larva (ion 371: F_1,48_ < 0.001, p = 0.99; ion 445: F_1,48_ = 0.018, p = 0.89), and third stage parasitoid larvae (ion 371: F_1,49_ = 0.006, p = 0.93; ion 445: F_1,49_ = 0.16, p = 0.69).

*Cyclosa fililineata* presented the same patterns, i.e., concentrations of 20E product ion 371 and ion 445 (pg/mL/mg) were higher in spiders parasitized by third stage larva (ion 371: F_1,24_ = 61.29, p < 0.001, Fig 2C, S1 Fig; ion 445: F_1,24_ = 78.68, p < 0.001, Fig 2D, S2 Fig) than in all other analyzed categories. All remaining categories were similar as follows: there were no differences in the concentrations of both 20E product ion 371 and ion 445 among unparasitized spiders, and second stage parasitoid larvae (ion 371: F_1,21_ = 0.45, p = 0.51; ion 445: F_1,21_ = 0.95, p = 0.34), spiders parasitized by second stage larva (ion 371: F_1,22_ = 0.68, p = 0.42; ion 445: F_1,22_ = 0.30, p = 0.58), and third stage parasitoid larvae (ion 371: F_1,23_ = 1.85, p = 0.19; ion 445: F_1,23_ = 1.19, p = 0.29).

## Discussion

We verified that the proximal mechanism that triggers the behavioral changes in web building in spiders carrying parasitoid larvae in the third stage of development involve increased 20E levels in the spider host's hemolymph. Our results reveal that the proximal mechanism that triggers this behavioral change in the host spiders activates similar web building behavior, as occurs in unparasitized, juvenile spiders just before ecdysis. The evidence for this conclusion was the observed similarities between molting and cocoon webs.

As far as we did not detect higher 20E levels in the third stage parasitoid larvae immediately before the larva's pupation, the mechanism that leads to increase in 20E levels within the host spider remains unknown. One reason for our results could be that when we sampled the third stage parasitoid larvae, the larvae had already produced and transferred 20E into the host spider. Evidence favouring this hypothesis is that when we sampled the third stage parasitoid larvae all of the host spiders had already built their cocoon webs. Therefore, the moment of 20E production might have preceded our sampling, and these higher 20E levels might have already been transferred to the host spider.

Another hypothesis for the proximate mechanism linking the parasitoid third stage larva to the increase in 20E levels in the host spider’s hemolymph is that the larvae do not produce 20E themselves, but, instead, produce a precursor chemical that is responsible for 20E synthesis in the spider host. Irrespective of the mechanism linking the parasitoid larva to the increase in 20E within its host, the injection of 20E or a precursor responsible for 20E synthesis into the spider host probably occurs directly while the larva is feeding on the spider.

Similarities between cocoon and molting webs of both studied species include a reduction in number of radii and the absence of sticky spirals (Fig 1). The same pattern was observed in *C*. *argenteoalba* [18] manipulated by *Reclinervellus nielseni* (Roman, 1923). In this study, a detailed comparison of web structures, building behavior, and silk spectral/tensile also showed that the few radii of both web types were decorated by many fibrous ultra violet-reflective threads. For *Leucauge volupis* (Keyserling, 1893) (Tetragnathidae) attacked by *Hymenoepimecis jordanensis* Loffredo & Penteado-Dias 2009, molting and cocoon webs share the presence of a three-dimensional component, a lower tangle, which might increase the stability of the horizontal web and is absent from normal webs constructed by adult spiders [22]. In addition, the cocoon webs of *Argiope trifasciata* (Forskäl, 1775) attacked by *Acrotaphus tibialis* (Cameron, 1886) included a three-dimensional structure similar to those observed in the molting webs of this species [34]. Interestingly, it is also possible that the manipulation of spiders by another taxonomical group, Diptera larvae of Acroceridae, involves the same hormone. These endoparasites of spiders also induce behavioral changes in web building behaviors that result in web designs similar to those of molting webs [35,36].

However, the generality of this mechanism cannot be confirmed from the observation of cocoon webs of other spiders, such as *Neottiura bimaculata* (Linnaeus, 1767) and *Theridion varians* Hahn, 1833 attacked by *Zatypota percontatoria* (Müller, 1776) in the Czech Republic [16]. The “cupula-like” structure built by parasitized *T*. *varians* and the denser web built by *N*. *bimaculata* are dissimilar to the structures constructed before molting. In fact, they resemble the web structures observed before overwintering in *T*. *varians* and before overwintering and oviposition in *N*. *bimaculata*. Thus, in these cases, behavioral modifications might be responses to the same substance produced and inoculated by the parasitoid larva [16]. This substance might activate innate behaviors that also occur during specific life-history periods of the hosts. Although ecdysteroids are also involved in the ovarian development of spiders [37], we would expect low levels of these hormones to be present before a period of reduced activity in cold winter conditions [24]. Other cases of cocoon webs presenting unique characteristics have been reported for *A*. *bifurca* [19], *Anelosimus* spp. [38], *Leucauge argyra* (Walckenaer, 1842) [10], *Leucauge mariana* (Keyserling, 1881) [34] and *Leucauge roseosignata* Mello-Leitão [39]. These characteristics of cocoon webs, differing from molting webs, show that other substances might be involved in the behavioral manipulation of spiders and that the mechanism described for *Cyclosa* species is not unique.

There is also variation in the way in which different parasitoid species modify the web design of the same host spider species. One such example is the case of *Araniella opisthographa* (Kulczynski, 1905) parasitized by *Polysphincta boops* Tschek, 1868, *Polyphincta tuberose* (Gravenhorst, 1829) and *Sinarachna pallipes* (Holmgren, 1860) wasps [17]. Although these differences might be related to different levels of 20E, it is possible that other parasitoid species produce other inducing substances, which can act alone or interact with 20E leading to variation in web architecture. Alternatively, as suggested by Eberhard [19], the action of the mature female wasp stabbing the host and injecting substances during or after the egg laying process might influence the sensitivity of the spider’s nervous system, making it sensitive to the higher levels of 20E later injected by the larvae.

We conclude that the seeming lack of control of parasitized *C*. *morretes* and *C*. *fililineata*, just before pupation could be induced by the anachronic activation of mechanisms that were originally involved in changing the spider's exoskeleton before maturation. This phenomenon could be achieved by increasing the levels of ecdysone during feeding by the parasitoid's larva on the spider host. Thus, the exposition to ecdysone probably induces innate behaviors performed during the construction of simplified resistant web architectures that are utilized to endure the molting period. These modifications increase web stability and protection for the spider during periods of higher susceptibility to predators and prevent the risk of webs collapsing.

## Supporting information

S1 Fig Representative chromatograms of ecdysone 371 ion product in *Cyclosa morretes* and *C. fililineata* spider hosts and respective second or third stage parasitoid larva (*Polysphincta janzeni* and *P.* sp. nr. *purcelli* wasps, respectively). Chromatograms were obtained by liquid chromatography mass spectrometry with the multiple reaction monitoring strategy detected with steroid-selective multiple reaction monitoring channels. The steroids were separated by reversed phase liquid chromatography with a complex gradient elution with acetonitrile/water. Data from third stage larvae correspond to individuals obtained after the construction of modified cocoon webs by their hosts.
(TIF)

S2 Fig Representative chromatograms of ecdysone 445 ion product in *Cyclosa morretes* and *C. fililineata* spider hosts and respective second or third stage parasitoid larva (*Polysphincta janzeni* and *P.* sp. nr. *purcelli* wasps, respectively). Chromatograms were obtained by liquid chromatography mass spectrometry with the multiple reaction monitoring strategy detected with steroid-selective multiple reaction monitoring channels. The steroids were separated by reversed phase liquid chromatography with a complex gradient elution with acetonitrile/water. Data from third stage larvae correspond to individuals obtained after the construction of modified cocoon webs by their hosts.
(TIF)

S1 File Data table for 20-OH-ecdysone concentration obtained in liquid chromatography mass spectrometry analysis of *Cyclosa morretes, C. fililineata* and its parasitoid wasps. (XLSX)
