# Correction: Proximate mechanism of behavioral manipulation of an orb-weaver spider host by a parasitoid wasp

**DOI:** 10.1371/journal.pone.0174146

**Published:** 2017-03-13

**Authors:** The PLOS ONE Staff

There are errors in the Funding section. The correct funding information is as follows:

TGK—Fundação de Amparo à Pesquisa e Inovação do Espírito Santo (Process 71277560/2015, TO 1020/2015) (https://fapes.es.gov.br); TGK, MOG, CFS—Conselho Nacional de Desenvolvimento Científico e Tecnológico (CNPq) (Grant numbers 140508/2012-0, 300295/2016-2,310032/2015-6, 306157/2014-4, 403733/2012-0, 445832/2014-2, 573802/2008-4) (http://www.cnpq.br); MOG—Fundação de Amparo à Pesquisa do Estado de Minas Gerais (Grant number APQ-02104-14, CRA-30058/12) (http://www.fapemig.br/pt-br); Coordenação de Aperfeiçoamento de Pessoal de Nível Superior (CAPES) (http://www.capes.gov.br); Financiadora de Estudos e Projetos (www.finep.gov.br); and Sistema Nacional de Laboratórios em Nanotecnologias (SisNANO)/Ministério da Ciência, Tecnologia e Informação (www.mcti.gov.br/sisnano).

The publisher apologizes for the error.
