# Supplementary figures and images for: Proximate mechanism of behavioral manipulation of an orb-weaver spider host by a parasitoid wasp
Source: PLoS One. 2017 Feb 3;12(2):e0171336. doi: 10.1371/journal.pone.0171336 (PMC5291528; doi:10.1371/journal.pone.0171336)

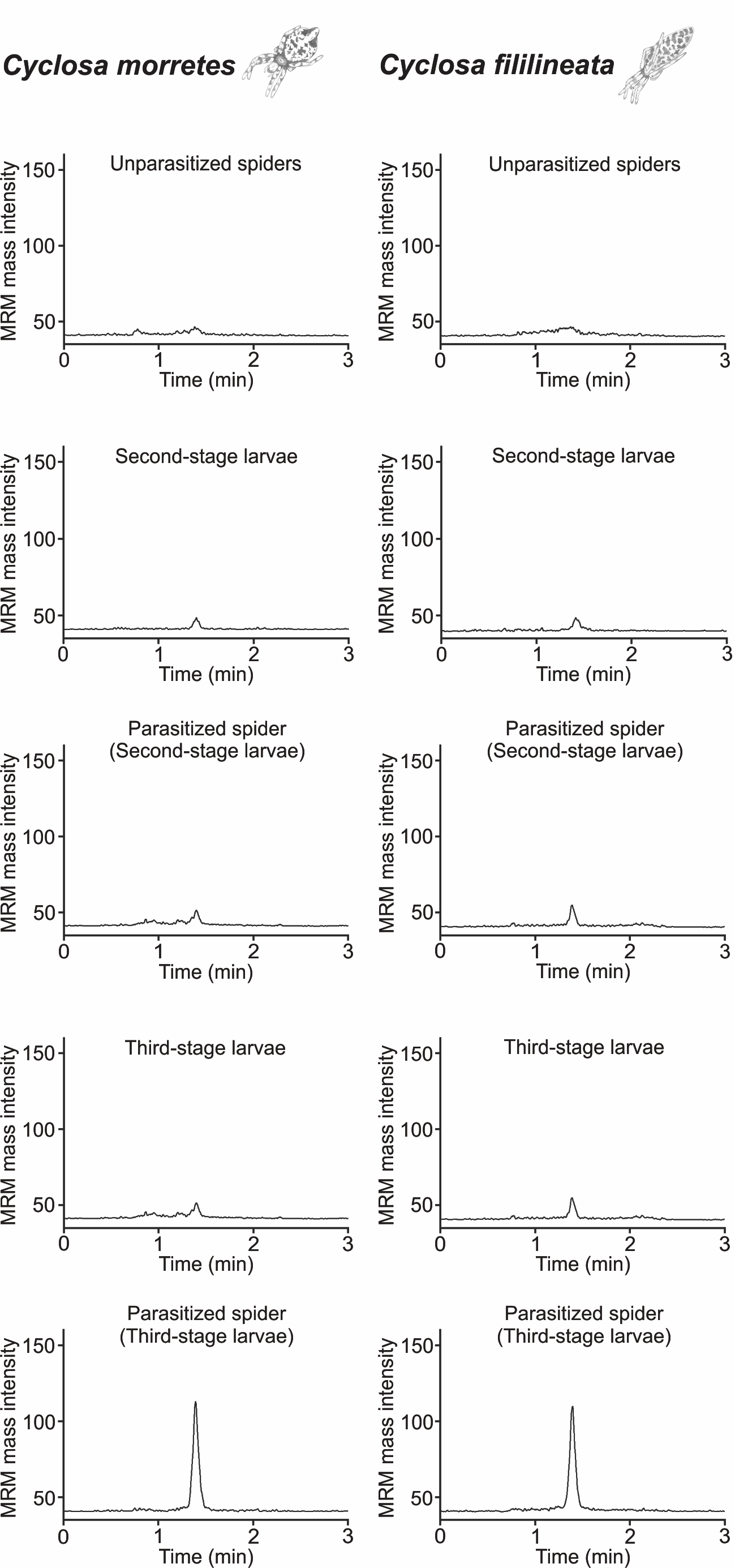

Supplement: S1 Fig — Chromatograms were obtained by liquid chromatography mass spectrometry with the multiple reaction monitoring strategy detected with steroid-selective multiple reaction monitoring channels. The steroids were separated by reversed phase liquid chromatography with a complex gradient elution with acetonitrile/water. Data from third stage larvae correspond to individuals obtained after the construction of modified cocoon webs by their hosts. (TIF) [file pone.0171336.s001.tif]

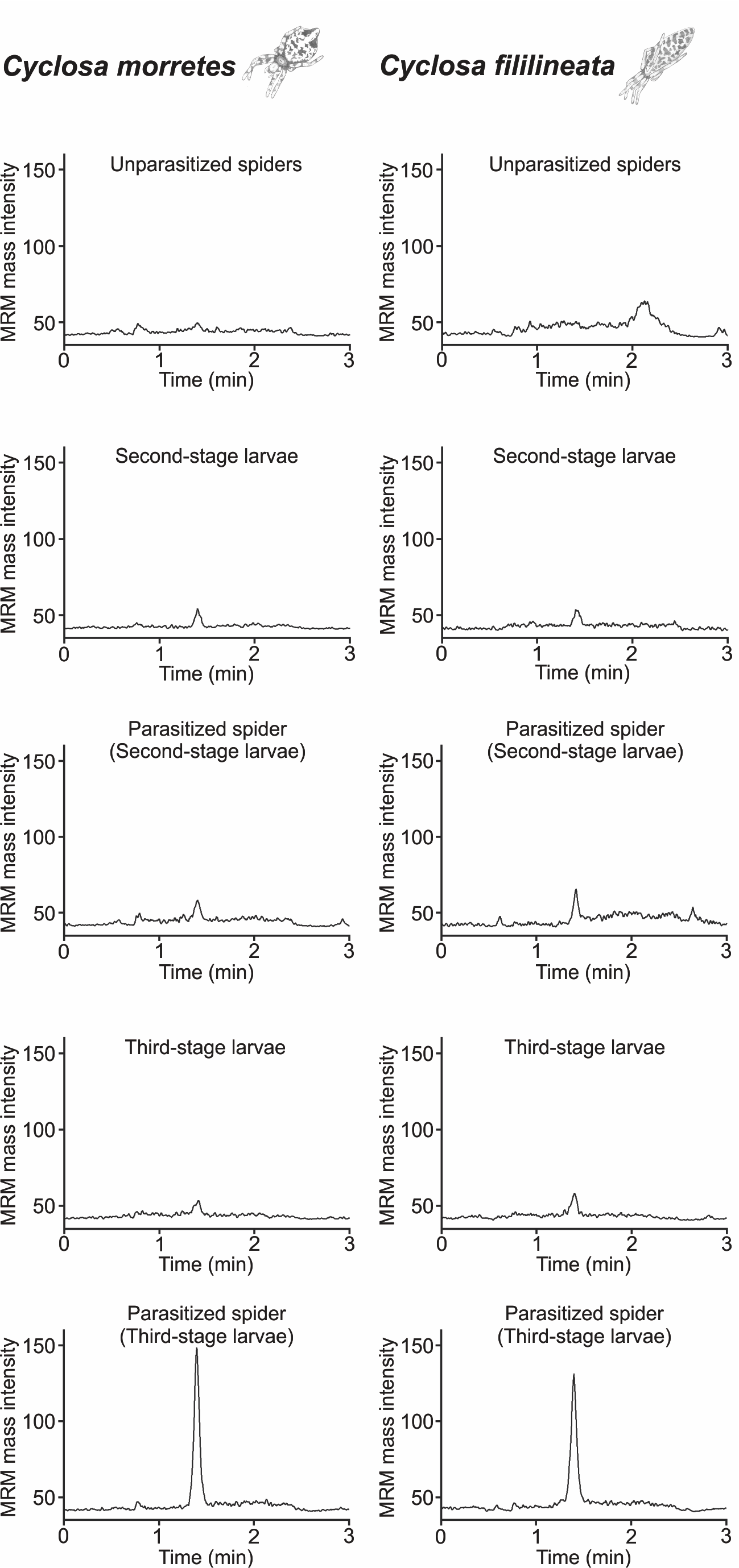

Supplement: S2 Fig — Chromatograms were obtained by liquid chromatography mass spectrometry with the multiple reaction monitoring strategy detected with steroid-selective multiple reaction monitoring channels. The steroids were separated by reversed phase liquid chromatography with a complex gradient elution with acetonitrile/water. Data from third stage larvae correspond to individuals obtained after the construction of modified cocoon webs by their hosts. (TIF) [file pone.0171336.s002.tif]
